# The utility of tumour markers in assessing the response to chemotherapy in advanced bladder cancer

**DOI:** 10.1054/bjoc.2000.1147

**Published:** 2000-05-18

**Authors:** A M Cook, R A Huddart, G Jay, A Norman, D P Dearnaley, A Horwich

**Affiliations:** The Royal Marsden NHS Trust and Institute of Cancer Research, Downs Road, Sutton, Surrey, SM2 5PT, UK

**Keywords:** bladder cancer, chemotherapy, tumour markers

## Abstract

In patients with advanced bladder cancer receiving chemotherapy, early assessment of response can avoid unnecessary toxicity. The aim of this study was to assess the role of tumour markers in monitoring response. Serum levels of one or more of markers β human chorionic gonadotrophin (βhCG), carcinoembryomic antigen (CEA), CA125 and CA19.9 were measured in 74 patients with advanced bladder cancer receiving chemotherapy from 1992 to 1997. Forty-three of 74 (58%) of patients had at least one raised marker (1.5 times upper limit of normal range). This was more common in patients with extra-pelvic disease than in those with disease confined to the pelvis (*P*= 0.002). Thirty-eight of 78 (49%) assessable patients had a radiological response. Neither clinical response (*P*= 0.81) nor survival (*P*= 0.16) differed between marker-negative and marker-positive patients. Clinical response was strongly related to marker response in the 35 comparable patients (*P*= 0.0001). No patient had a clinical response without response of at least one marker. Ninety per cent of patients who achieved a marker response had done so by 8 weeks. Monitoring of tumour markers in patients with advanced bladder cancer can help predict the response to chemotherapy. © 2000 Cancer Research Campaign


British Journal of Cancer (2000) **82**(12), 1952–1957
© 2000 Cancer Research Campaign
doi: 10.1054/ bjoc.2000.1147, available online at http://www.idealibrary.com on IDEAL®

### The utility of tumour markers in assessing the response to chemotherapy in advanced bladder cancer

**AM Cook, RA Huddart, G Jay, A Norman, DP Dearnaley and A Horwich**

The Royal Marsden NHS Trust and Institute of Cancer Research, Downs Road, Sutton, Surrey SM2 5PT UK

**Summary** In patients with advanced bladder cancer receiving chemotherapy, early assessment of response can avoid unnecessary toxicity. The aim of this study was to assess the role of tumour markers in monitoring response. Serum levels of one or more of markers β human chorionic gonadotrophin (βhCG), carcinoembryomic antigen (CEA), CA125 and CA19.9 were measured in 74 patients with advanced bladder cancer receiving chemotherapy from 1992 to 1997. Forty-three of 74 (58%) of patients had at least one raised marker (1.5 times upper limit of normal range). This was more common in patients with extra-pelvic disease than in those with disease confined to the pelvis ($P = 0.002$). Thirty-eight of 78 (49%) assessable patients had a radiological response. Neither clinical response ($P = 0.81$) nor survival ($P = 0.16$) differed between marker-negative and marker-positive patients. Clinical response was strongly related to marker response in the 35 comparable patients ($P = 0.0001$). No patient had a clinical response without response of at least one marker. Ninety per cent of patients who achieved a marker response had done so by 8 weeks. Monitoring of tumour markers in patients with advanced bladder cancer can help predict the response to chemotherapy. © 2000 Cancer Research Campaign

**Keywords**: bladder cancer; chemotherapy; tumour markers

The treatment of advanced bladder cancer frequently involves the use of intensive chemotherapy. Recent randomized trials suggest that the use of regimens such as M-VAC or CMV improves disease-free and overall survival (Logothetis et al, 1990; Loehrer et al, 1992; Fosså et al, 1996). A group of good prognosis patients with good performance status and disease confined to the pelvis may have a 5-year survival rate of up to 28% (Fosså et al, 1996). However, the median survival of patients with metastatic disease who do not have these good prognostic features is only 10 months (Fosså et al, 1996). Patients are frequently elderly, frail and may have impaired renal function, and toxicity is significant, with 4% treatment-related mortality (Sternberg et al, 1988; Loehrer et al, 1992; Fosså et al, 1996). Only patients who have a good response to chemotherapy seem to gain significant survival benefit (Fosså et al, 1996), so early assessment of response is desirable to avoid unnecessary toxicity in patients who are not responding to treatment and may have a limited life expectancy. In several tumour types (for example, testicular germ cell tumours and prostate cancer) tumour markers, when raised, can play a useful role in monitoring response to treatment and have resulted in important improvements in management.

In bladder cancer several tumour markers have been reported as being raised including β human chorionic gonadotrophin (βhCG), carcinoembryomic antigen (CEA), CA 19.9, CA 125 and tissue polypeptide antigen (TPA). βhCG is the most widely reported and has been found both in the serum and urine (Dexeus et al, 1986; Iles et al, 1989, 1996; Williams et al, 1990; McLoughlin et al, 1991; Marcillac et al 1992, 1993) and on immunohistochemical staining (Wurzel et al, 1987; Moutzouris et al, 1993) in many patients with bladder cancer. Iles et al (1989) report that serum levels of βhCG were raised in 76% of patients with widespread metastases but only 3% with disease confined to the pelvis (Iles et al, 1989). Other estimates of the frequency with which βhCG is elevated vary from 14% (McLoughlin et al, 1991) to 47% (Marcillac et al, 1992). In preliminary work in patients with locally advanced/metastatic disease, who have significantly raised tumour markers, marker levels have been shown to correlate with the clinical course of the disease (Marcillac et al, 1992) and response to chemotherapy (Dexeus et al, 1986; Marcillac et al, 1993).

Other markers are less commonly described, although Dexeus et al (1986) found CEA to be raised in 23% of patients with advanced bladder cancer referred for chemotherapy the level correlating with clinical response (Dexeus et al, 1986). CA 19.9 has been found to be raised, in up to 40% (8/20) patients with bladder cancer (Abel et al, 1987; Pectasides et al, 1996). CA125 was reported as raised in one patient with adenocarcinoma of the urachus, which fell with response to chemotherapy (Guarnaccia et al, 1991). Recently TPA has also been reported to be a sensitive marker of bladder cancer (particularly advanced or metastatic disease) (Maulard Durdux et al, 1997) and may be useful in monitoring response to treatment (Schmidt et al, 1992; Pectasides et al, 1996).

The aim of this study was to assess how often certain tumour markers were raised in patients receiving chemotherapy for locally advanced or metastatic bladder cancer and to determine whether they could have a role in monitoring the response to treatment. It was previously decided to concentrate on the markers shown to be raised for which tests were routinely available at our Institution (βhCG, CEA, CA19.9 and CA125).

*Received 22 March 1999*
*Accepted 25 January 2000*

*Correspondence to:* RA Huddart

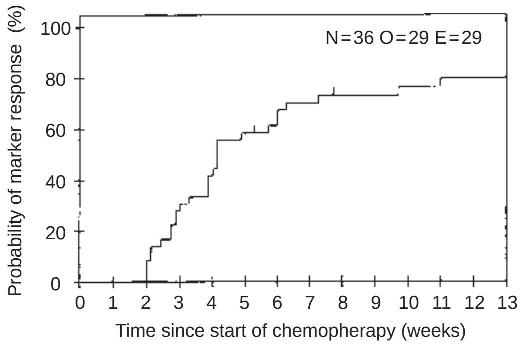

**Figure 1** Time to partial response of any marker

### PATIENTS AND METHODS

From July 1992 to July 1997 serum levels of tumour markers were measured in 74 of 78 patients with locally advanced or metastatic bladder cancer referred for consideration of chemotherapy.

The most common regimen used was M-VAC (Sternberg et al, 1988) (methotrexate 30 mg $m^{-2}$ on days 1, 15, 22, vinblastine 3 mg $m^{-2}$ on days 1, 15, 22, doxorubicin 30 mg $m^{-2}$ on day 1, cisplatin 70 mg $m^{-2}$ on day 1). Twenty-three patients had modified regimens. Fifteen patients were given carboplatin instead of cisplatin, methotrexate was omitted in three patients (with pleural effusions or ascites), one patient did not receive doxorubicin because of impaired hepatic function and vinblastine was omitted in two patients. Two patients were treated with carboplatin and methotrexate only. The median number of cycles given was three (range 1–6).

At the start of the study only βhCG and CEA were measured routinely. From November 1994 CA19.9 was added, but CA125 was only routinely checked from May 1996. Marker analysis was performed on a Roche Cobas Core analyser (Roche Diagnostics) until December 1996 and thereafter on an Abbott Axsym analyser (Abbott Diagnostic Laboratories). Normal ranges as per manufacturing reccomendations (βhCG < 4 IU $L^{-1}$, CEA < 5 µg $ml^{-1}$, CA19.9 < 35 U $ml^{-1}$ and CA125 < 35 U $ml^{-1}$). Levels above the normal range were noted but only those of 1.5 times the upper limit of the normal range were classified as being raised. Also 1.5 times the upper limit was chosen in line with some systems of prognostic grouping where a marker raised more than minimally to 1.5 times is considered abnormal (International Germ Cell Cancer Collaborative Group, 1997). It was aimed to repeat any markers that were raised prior to chemotherapy on day 1 of each cycle of chemotherapy. Those that were not raised prior to treatment were not repeated as they were unlikely to be raised and it was not considered to be cost-effective.

Sites of disease were recorded prior to chemotherapy. Patients with pelvic nodal or locally advanced disease only were classified as having pelvic disease and all others were considered to have extra-pelvic disease. Clinical response was assessed according to radiological findings by a consultant radiologist after 2, 4 and 6 cycles of chemotherapy. A complete response (CR) representing no radiological evidence of disease and a partial response (PR) a considerable reduction in disease volume equivalent to 50% or more. Stable or progressive disease was classified as no response. Marker response was defined as a 50% fall in marker levels and complete response as return of markers to normal levels.

**Table 1** Patient characteristics

| Patient characteristic | Number of patients |
|---|---|
| Sex | |
| Male | 64 |
| Female | 14 |
| Age | |
| Median 62 years (36–75) | |
| Sites of disease | |
| Pelvic | 29 |
| Bladder alone | 7 |
| Pelvic lymph nodes+/–bladder | 22 |
| Extra-pelvic | 49 |
| Para-aortic nodes | 18 |
| Lung | 16 |
| Liver | 7 |
| Bone | 11 |
| Other[a] | 25 |
| Histology | |
| Transitional cell | 73 |
| Adenocarcinoma | 1 |
| Squamous | 0 |
| Undifferentiated | 4 |

[a] Including psoas muscle, mediastinal lymph nodes, pleural effusions.

Individual marker responses were compared with clinical response, but in cases where more than one marker was raised and there had been a differing response between the markers, the response in the marker showing the greatest and that showing the poorest response were also recorded and compared with clinical response. These were recorded as ‘best’ and ‘worst’ marker response respectively.

### Statistical analysis

Categorical data were examined using the $\chi^2$ test with Fisher’s Exact test (one-tail, *P*-values) where appropriate. Survival analysis was performed using the methods of Kaplan and Meier and differences in survival curves examined using the log-rank test. Figure 1 was generated using the methods of Kaplan and Meier but any marker response (partial or complete) was considered as the event.

### RESULTS

There was a total of 78 patients who received chemotherapy for bladder cancer during this period. Patient characteristics are given in Table 1.

### Frequency of raised markers in patients with bladder cancer

One or more pre-treatment tumour marker was checked in 74 of the 78 patients. Overall, 43 of the 74 (58%) patients had at least one marker more than 1.5 times the upper limit of the normal range. Fifty-two of 74 (70%) had elevation of at least one marker above the normal range.

At least one tumour marker was more frequently raised in patients known to have extra-pelvic disease (34/47, 72%) than in those who had disease confined to the pelvis (9/27, 33%) ($P$ = 0.001). This was not clearly demonstrated when analysing individual markers as the numbers were too small.

© 2000 Cancer Research Campaign

**Table 2** Clinical response in patients with negative markers compared to those with one or more positive markers

| | Clinical response | | | |
|---|---|---|---|---|
| **No. raised markers** | **Complete response** | **Partial response** | **No response** | **Total** |
| 0 | 3 | 12 | 16 | 31 |
| 1 or more | 7 | 15 | 21 | 43 |
| Not known | 0 | 1 | 3 | 4 |
| Total | 10 | 28 | 40 | 78 |

Chi-squared test of responders (with complete or partial response) compared with non-responders for patients with positive or negative markers $P = 0.81$.

**Table 3** Clinical response compared to marker response in evaluable patients

| | Clinical response | | | |
|---|---|---|---|---|
| | **Complete response** | **Partial response** | **No response** | **Total** |
| βhCG response[a] | | | | |
| Complete | 2 | 9 | 4 | 15 |
| Partial | 1 | 1 | 1 | 3 |
| No response | 0 | 0 | 1 | 1 |
| Total | 3 | 10 | 6 | 19 |
| CEA response[b] | | | | |
| Complete | 2 | 1 | 0 | 3 |
| Partial | 2 | 1 | 2 | 5 |
| No response | 0 | 1 | 5 | 6 |
| Total | 4 | 3 | 7 | 14 |
| Ca19.9 response[c] | | | | |
| Complete | 1 | 1 | 1 | 3 |
| Partial | 0 | 4 | 1 | 5 |
| No response | 0 | 0 | 5 | 5 |
| Total | 1 | 5 | 7 | 13 |
| Ca125 response | | | | |
| Complete | 0 | 1 | 1 | 2 |
| Partial | 0 | 0 | 0 | 0 |
| No response | 0 | 0 | 0 | 0 |
| Total | 0 | 1 | 1 | 2 |

[a] Fisher's exact test of responders (complete or partial) vs non-responders $P = 0.32$ (one-tail). [b] Fisher's exact test (one tail) of responders vs non-responders $P = 0.052$. [c] Fisher's exact test (one-tail) of responders vs non-responders $P = 0.17$.

Twenty-one of 71 (30%) of patients had a significantly raised βhCG, 16 of 67 (24%) a raised CEA, 19 of 44 (43%) a raised CA19.9 and three of 21 (14%) a raised CA125. Fourteen of the 43 patients with raised markers had more than one marker raised (12 had two raised markers and two had three) with 29 of 43 having one only.

The median values (range), of markers before treatment were βhCG (median 81, range 7–3303), CEA (median 40, range 10–1280), CA19.9 (median 236, range 59–10920) and CA125 (median 142.5, range 128–157). Mean levels (range), after treatment were βhCG (mean 2, range 2–58), CEA (mean 11, range 1–272), CA19.9 (mean 121, range 6–8730) and CA125 (mean 23, range 16–30).

### Response

Of the 78 patients treated, seven died early in their treatment, five after only one cycle of chemotherapy and two just after the second cycle (despite early suggestions of response). These have all been classified as non-responders.

The overall clinical response rate was 38 of 78 patients (49%), of which ten of 78 (13%) had a complete response and 28 of 78 (36%) had a partial response. For patients with extra-pelvic disease overall response rate was 22 of 49 (44.9%, confidence interval (CI) 30.7–59.8) and for pelvic disease 16 of 29 (55.2%, CI 35.7–73.6)). The difference was not significant ($P = 0.38$). There was no significant difference in response rate in all patients with one or more positive markers compared with that in patients with negative markers ($P = 0.81$) (see Table 2), suggesting that the presence of a raised pretreatment marker to > 1.5 times the normal range is not a useful prognostic factor for response in patients with metastatic disease.

The proportion of patients with a marker response varied according to the individual marker. In patients with a raised βhCG, 18 of 19 achieved a response, while only eight of 14 patients with a raised CEA and eight of 14 patients with a raised CA 19.9 responded.

Table 3 shows the clinical response compared to the marker response (in evaluable patients) for each tumour marker in turn. The numbers were small; however, there was a suggestion that marker response was related to clinical response, especially with

 © 2000 Cancer Research Campaign

**Table 4** Clinical response compared to 'worst' marker response in evaluable patients

| | Clinical response | | | |
|---|---|---|---|---|
| | **Complete response** | **Partial response** | **No response** | **Total** |
| 'Worst' marker response | | | | |
| Complete | 3 | 9 | 4 | 16 |
| Partial | 3 | 4 | 1 | 8 |
| No response | 0 | 1 | 11 | 12 |
| Total | 6 | 14 | 16 | 36 |
| 'Best' marker response | | | | |
| Complete | 4 | 12 | 6 | 22 |
| Partial | 2 | 2 | 3 | 7 |
| No response | 0 | 0 | 7 | 7 |
| Total | 6 | 14 | 16 | 36 |

[a] Fisher's exact test (one tail) of responders vs non-responders $P < 0.0001$; Sensitivity 95%, Specificity 69%.[b] Fisher's exact test (one tail) of responders vs non-responders $P = 0.0017$; Sensitivity 100%, Specificity 44%.

CEA ($P$ = 0.051) and CA19.9 ($P$ = 0.17). It appears that βhCG may overestimate response. Tables 4A and 4B show that the clinical response is strongly related to both the 'worst' (that showing the least response where more than one marker was raised) ($P$ < 0.0001), and 'best' (that showing the greatest) marker response ($P$ = 0.0017). Figure 1 shows the probability of achieving a partial marker response. There was a 46% chance of responding by 4 weeks, with a 72% chance of responding by 8 weeks. Only three of 29 patients who did have a partial marker response had not responded by 8 weeks, thus 90% of patients who eventually did have a partial marker response had done so by 8 weeks. The three patients achieving a marker response after this time did so at 68, 77 and 114 days, but this apparent delay may be misleading. In all three the markers had fallen early in treatment but were not checked at 8 weeks and by the time they were subsequently checked a response had been achieved. Of the 29 patients who eventually achieved a marker response, markers had been checked after 4 weeks in 23. In all of these the responding marker had fallen by at least 35% by 4 weeks. The median time to achieve at least a partial marker response was 29 days (range 14–114).

If the 'worst' marker response is considered it can be seen that in 18 of 36 patients the marker response was the same as the clinical response and in 14 of 36 patients the marker response was better than the clinical response. In five cases serum markers predicted response but clinical response was not demonstrated. There was, however, only one patient who had no marker response but did have a clinical response. In this patient the CEA was initially only slightly raised at 10 and it fell to 8 during treatment. This patient also had a more markedly raised βhCG at 188, which fell substantially to 5 during treatment. Overall the sensitivity was 95% and specificity 69%.

When considering the 'best' marker responses, it is notable that nine patients had a marker response with no definite clinical response. In one of these patients there was a differential response with lung metastases resolving with treatment (coinciding with a fall in βhCG from 7 to 2) but a pelvic mass subsequently increasing in size. In five of these nine patients another marker was also raised, and in four of these the other marker showed no response. Thus, the sensitivity was 100%, but specificity was reduced to 44%.

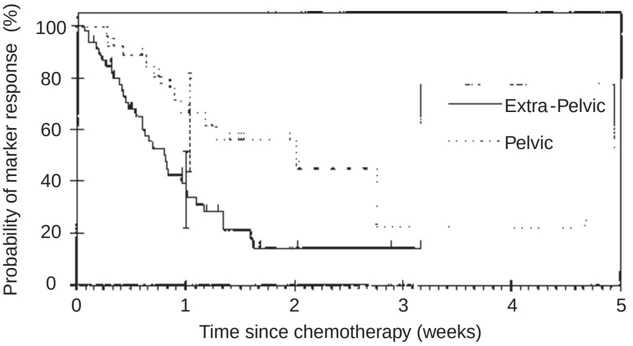

**Figure 2** Survival by site of disease

### Survival

As expected, overall survival was poor but the patients with disease confined to the pelvis (median survival 24 months) survived significantly longer than those with extra-pelvic disease (median survival 9 months) ($P$ = 0.006) (Figure 2).

There was no difference in survival between marker positive and marker negative patients ($P$ = 0.155) (Figure 3). Small numbers made it difficult to compare survival in patients who achieved a marker response with those who did not achieve a marker response.

### DISCUSSION

A high proportion of patients with metastatic bladder cancer have one or more raised tumour markers. This is especially true for patients with extra-pelvic disease in whom, in this series, 70% had at least one marker significantly raised. By nature of the evolution of this project not all patients had all markers checked so this may represent the lower end of the expected rate. The increased frequency of marker elevation in extra-pelvic disease suggests that the presence of raised tumour markers is related to tumour bulk.

In this study, however, there was no definite evidence of raised pretreatment tumour markers being a poor prognostic factor, in

© 2000 Cancer Research Campaign

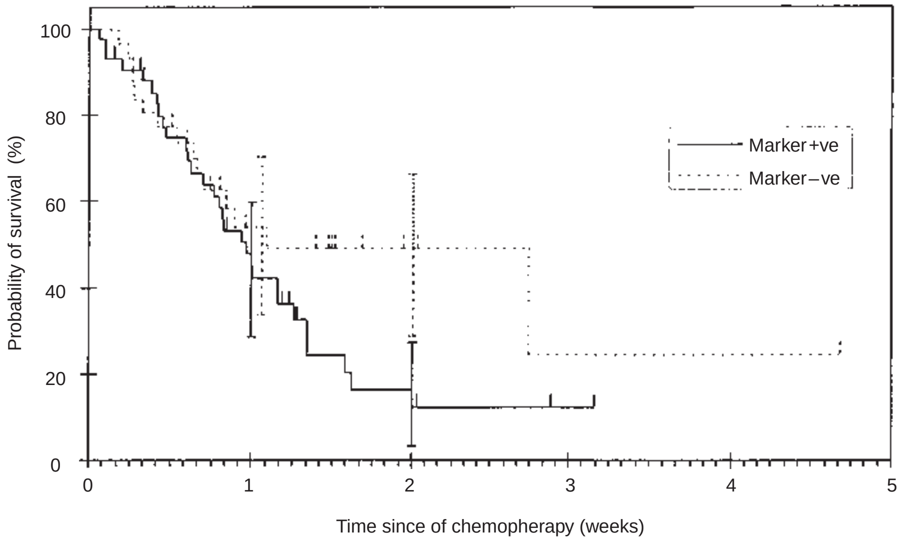

**Figure 3** Prechemotherapy markers raised vs not raised patients with metastatic disease. However, the study only has limited power and this result may be confounded by some earlier patients only having one, or more commonly two, markers measured and thus not representing truly marker-negative patients. Previous studies (Dexeus et al, 1986; McLoughlin et al, 1991) which have found βhCG to be a poor prognostic factor have been in patients with localized disease. Iles et al (1996) investigated urinary hCG levels. In this study urinary βhCG levels in patients with T2–T4 disease were significantly associated with development of metastases and survival. Moutzouridis et al (1993) looked at immunohistochemistry of biopsies from patient with apparently localized bladder cancer and noted a significant difference in response to radiotherapy with βhCG-positive tumours responding poorly and these patients tended to have a worse survival.

There is a clear relationship between marker response and clinical response. It might be expected that a raised tumour marker would respond more rapidly to treatment than radiological appearances. The evidence presented here suggests that if a marker response is to occur it occurs quickly, with 90% of responders having had a 50% fall by 2 months and some degree of fall by 4 weeks. As markers were largely only taken when patients attended for treatment this, if anything, represents a 'worst' case situation and that marker response could be detected as early as 4–6 weeks after treatment is commenced. It is also clear from our data that in the presence of stable or rising marker levels radiological response is unlikely and would suggest that alternative treatment strategies should be pursued.

The converse is not always true with a proportion of patients achieving a marker response without clinical evidence of a partial response. This was particularly noticeable with βhCG, where five of 19 patients had a marker response but no clinical response. Similar effects have been seen in prostate-specific antigen levels in prostate cancer following chemotherapy and could be due to a number of mechanisms including clonal selection of non marker secreting cells or modification of marker secretions by tumour cells in response to chemotherapy.

The specificity of the marker response was increased when more than one marker was followed. The 'worst' marker response, in particular, was very closely related to clinical response. A policy of testing initially for all four tumour markers mentioned (and then following only the raised marker) increases the chances of finding a raised marker and, where more than one is raised, the accuracy with which response can be predicted. We are now using this policy as part of our routine management for patients receiving palliative chemotherapy for bladder cancer. Response to chemotherapy is often difficult to assess and if at least one marker is raised pre-treatment but does not fall with treatment and unless there was often clear indication of response we would stop chemotherapy after two cycles, so saving unnecessary toxicity for the patient and cost for the hospital.

Recently TPA has also been used as a marker for monitoring therapeutic efficacy in bladder cancer (Pectasides et al, 1996), including after MVEC chemotherapy (Schmidt et al, 1992). It was found to be a sensitive marker (although sensitivity depends on the definition of the upper limit of the normal range) (Maulard Durdux et al, 1997) and complete marker response correlated well with complete clinical response. If this assay is readily available it may be useful combined with the markers used in this study or possibly further markers to increase the sensitivity of this approach.

In summary, these findings suggest that monitoring a panel of currently available tumour markers in bladder cancer can help predict the clinical response of patients to chemotherapy. Further investigation of these markers, with other newer ones, in advanced bladder cancer is indicated to obtain more information on the relative worth of individual markers and more details on the characteristics of marker response.

### ACKNOWLEDGEMENTS

This work was undertaken by The Royal Marsden NHS Trust who received a proportion of its funding from the NHS Executive; the views expressed in this publication are those of the authors and not necessarily those of the NHS Executive. This work was also supported by the Institute of Cancer Research and the Bob Champion Trust and the Cancer Research Campaign.

*© 2000 Cancer Research Campaign*

### REFERENCES

Abel PD, Cornell C, Buamah PK and Williams G (1987) Assessment of serum CA19.9 as a tumour marker in patients with carcinoma of the bladder and prostate. *Br J Urol* **59**: 427–429

Dexeus F, Logothetis C, Hossan E and Samuels ML (1986) Carcinoembryonic antigen and beta-human chorionic gonadotropin as serum markers for advanced urothelial malignancies. *J Urol* **136**: 403–407

Fosså SD, Sternberg C, Scher HI, Theodore CH, Mead B, Dearnaley D, Roberts JT and Skovlund E (1996) Survival of patients with advanced urothelial cancer treated with cisplatin-based chemotherapy. *Br J Cancer* **74**: 1655–1659

Guarnaccia S, Pais V, Grous J and Spirito N (1991) Adenocarcinoma of the urachus associated with elevated levels of CA 125. *J Urol* **145**: 140–141

Iles RK, Jenkins BJ, Oliver RT, Blandy JP and Chard T (1989) Beta human chorionic gonadotrophin in serum and urine. A marker for metastatic urothelial cancer. *Br J Urol* **64**: 241–244

Iles RK, Persad R, Trivedi M, Sharma KB, Dickinson A, Smith P and Chard T (1996) Urinary concentration of human chorionic gonadotrophin and its fragments as a prognostic marker in bladder cancer. *Br J Urol* **77**: 61–69

International Germ Cell Cancer Collaborative Group (1997) International Germ Cell Consensus Classification: A prognostic factor-based staging system for metastatic germ cell cancers. *J Clin Oncol* **15**: 594–603

Loehrer PJ, Sr, Einhorn LH, Elson PJ, Crawford ED, Kuebler P, Tannock I, Raghavan D, Stuart Harris R, Sarosdy MF, Lowe BA and et al. (1992) A randomized comparison of cisplatin alone or in combination with methotrexate, vinblastine, and doxorubicin in patients with metastatic urothelial carcinoma: a cooperative group study [published erratum appears in *J Clin Oncol* 1993 **11**: 384]. *J Clin Oncol* **10**: 1066–1073

Logothetis CJ, Dexeus FH, Finn L, Sella A, Amato RJ, Ayala AG and Kilbourn RG (1990) A prospective randomized trial comparing MVAC and CISCA chemotherapy for patients with metastatic urothelial tumors. *J Clin Oncol* **8**: 1050–1055

Marcillac I, Troalen F, Bidart JM, Ghillani P, Ribrag V, Escudier B, Malassagne B, Droz JP, Lhomme C, Rougier P and et al. (1992) Free human chorionic gonadotropin beta subunit in gonadal and nongonadal neoplasms. *Cancer Res* **52**: 3901–3907

Marcillac I, Cottu P, Theodore C, Terrier Lacombe MJ, Bellet D and Droz JP (1993) Free hCG-beta subunit as tumour marker in urothelial cancer [letter]. *Lancet* **341**: 1354–1355

Maulard Durdux C, Toubert ME, Hennequin C and Housset M (1997) Serum tissue polypeptide antigen in bladder cancer as a tumor marker: a prospective study. *J Clin Oncol* **15**: 3446–3450

McLoughlin J, Pepera T, Bridger J and Williams G (1991) Serum and urinary levels of beta human chorionic gonadotrophin in patients with transitional cell carcinoma [see comments]. *Br J Cancer* **63**: 822–824

Moutzouris G, Yannopoulos D, Barbatis C, Zaharof A and Theodorou C (1993) Is beta-human chorionic gonadotrophin production by transitional cell carcinoma of the bladder a marker of aggressive disease and resistance to radiotherapy? *Br J Urol* **72**: 907–909

Pectasides D, Bafaloucos D, Antoniou F, Gogou L, Economides N, Varthalitis J, Dimitriades M, Kosmidis P and Athanassiou A (1996) TPA, TATI, CEA, AFP, beta-hCG, PSA, SCC, and CA 19–9 for monitoring transitional cell carcinoma of the bladder. *Am J Clin Oncol* **19**: 271–277

Schmidt A, Bub P, Ruther U and Eisenberger F (1992) Tissue polypeptide antigen for monitoring of advanced bladder cancer after MVEC chemotherapy. *Eur Urol* **1**: 10–12

Sternberg CN, Yagoda A, Scher HI, Watson RC, Herr HW, Morse MJ, Sogani PC, Vaughan ED, Jr, Bander N, Weiselberg LR and et al. (1988) M-VAC (methotrexate, vinblastine, doxorubicin and cisplatin) for advanced transitional cell carcinoma of the urothelium. *J Urol* **139**: 461–469

Williams G, Colbeck RA and Crawford SM (1990) Treatment of bladder carcinoma using a germ cell chemotherapy protocol. *Br J Urol* **65**: 473–477

Wurzel RS, Yamase HT and Nieh PT (1987) Ectopic production of human chorionic gonadotropin by poorly differentiated transitional cell tumors of the urinary tract. *J Urol* **137**: 502–504

*© 2000 Cancer Research Campaign*
